# *Serendipita* Species Trigger Cultivar-Specific Responses to *Fusarium* Wilt in Tomato

**DOI:** 10.3390/agronomy9100595

**Published:** 2019-09-28

**Authors:** Negar Ghezel Sefloo, Krzysztof Wieczorek, Siegrid Steinkellner, Karin Hage-Ahmed

**Affiliations:** Institute of Plant Protection, Department of Crop Sciences, University of Natural Resources and Life Sciences, Vienna, 3430 Tulln, Austria

**Keywords:** *Fusarium oxysporum* f. sp. *lycopersici*, tomato, *Serendipita herbamans*, *Serendipita williamsii*, *Serendipita vermifera*, *Serendipita indica*, biological control, Micro-Tom, fungal endophytes

## Abstract

The endophytic fungi *Serendipita indica* and *S. vermifera* have recently gained increasing attention due to their beneficial effects on plant growth and plant health. Little is known about other species, such as *S. williamsii* and *S. herbamans*. To test their biocontrol and growth-promoting potential, susceptible and tolerant tomato cultivars (Kremser Perle and Micro-Tom, respectively) were inoculated with *S. williamsii, S. herbamans, S. indica*, or *S. vermifera* and challenged with the soilborne pathogen *Fusarium oxysporum* f. sp. *lycopersici* (*Fol*) in greenhouse experiments. Furthermore, in vitro assays on the direct inhibitory effects of *Serendipita* spp. against *Fol* were performed. Negative effects of *Fol* on phenological growth in the susceptible cultivar were alleviated by all four applied *Serendipita* spp. Apart from these similar effects on biometric parameters, disease incidence was only reduced by *S. herbamans* and *S. vermifera*. In the tolerant cultivar, disease parameters remained unaffected although shoot dry mass was negatively affected by *S. vermifera*. Direct effects of *Serendipita* spp. against *Fol* were not evident in the in vitro assays indicating an indirect effect via the host plant. Our results highlight the importance of identifying cultivar-specific effects in pathogen–endophyte–plant interactions to determine the most beneficial combinations.

## 1 Introduction

Fungal endophytes have recently gained increasing attention due to their growth-promoting and bioprotective properties. The testing and exploitation of these fungi might offer new sources for biological control and disease management strategies. One group of endophytic fungi belonging to the family *Serendipitaceae* (formerly *Sebacinales* group B) includes such promising candidates and is able to associate with the roots of various plant species [1]. To date, the most well-studied members are *Serendipita indica* (syn. *Piriformospora indica*) and *S. vermifera* (syn. *Sebacina vermifera*), representing only a small selection of the widespread fungi [1–3].

*S. indica* was first isolated from a *Funneliformis mosseae* (syn. *Glomus mosseae*) spore that originated from the rhizosphere of the woody shrubs *Prosopis juliflora* and *Zizyphus nummularia* growing in desert soil in northwestern India [4]. *S. indica* associates readily with a large number of plant species and can be easily cultured on common artificial media. In previous studies, this fungus was shown to promote plant growth and seed production in a wide range of host plants including wheat and barley [5,6], maize [7], tobacco [8], soybean [9], and *Arabidopsis* [10]. Studies on biotic stress alleviation as a response to *S. indica* root colonization have been mostly conducted with barley, wheat, and *Arabidopsis* as host plants. In these studies, disease control against *Blumeria graminis* f. sp. *hordei* [11], *Fusarium graminearum* [12] and *F. culmorum* in barley [5,13], and *B. graminis* f. sp. *tritici, F. culmorum* and *Pseudocercosporella herpotrichoides* in wheat [6] was shown. Disease control in tomato was reported against *F. oxysporum* [14], *Verticillium dahliae*, and Pepino mosaic virus (PepMV), depending on the environmental conditions [15].

*S. vermifera* was isolated from the roots of the orchid *Cyrtostylis reniformis* in Australia [16]. Several reports have indicated that *S. vermifera* is capable of promoting plant growth in a variety of plants [11,17–20]. Some studies on *S. vermifera* were conducted with respect to its activity as a biocontrol agent against soilborne pathogens including *Gaeumannomyces graminis* var. *tritici* causing take-all disease in wheat [21], *F. oxysporum* f. sp. *lentis* causing *Fusarium* wilt in lentil [22], and the leaf pathogen *B. graminis* f. sp. *hordei* in barley [11]. Only recently, it was shown that *S. vermifera* acted against *Bipolaris sorokiniana* in the rhizosphere of barley by reducing the virulence potential of the pathogen prior to host plant infection [23].

In spite of their potential, using exotic microorganisms such as *S. indica* and *S. vermifera* in European agricultural systems might be critical with regard to local fungal endophytic populations and unknown host ranges [24]. Furthermore, there is evidence from molecular data that the prevalence of taxa from *Sebacinales* is increased in wheat roots from organically managed compared to conventionally managed fields in Switzerland [2], indicating a still hidden source of species in European soils. Consequently, there is a strong need for testing and analyzing the potential of local species and isolates.

Recently, *S. herbamans* was isolated from the roots of *Bistorta vivipara* in Germany [25] and proved to be a promising candidate for possible application in Europe. Another candidate is *S. williamsii* (syn. *Piriformospora williamsii*, ex multinucleate rhizoctonia), which was isolated from a pot culture of *Rhizophagus fasciculatus* (syn. *Glomus fasciculatum* originated from soil from Rothamsted Research, UK) associated with clover roots [26,27]. *S. williamsii* did not show any growth promotion of *Arabidopsis* in vitro [28] but increased plant biomass when combined with arbuscular mycorrhizal fungi (AMF) in a greenhouse experiment [26]. However, to date, no information is available on the bioprotective potential of *S. herbamans* and *S. williamsii*, which might be new biocontrol sources against soilborne pathogens.

*Fusarium* wilt of tomato is caused by the soilborne pathogen *F. oxysporum* f. sp. *lycopersici* (*Fol*) and is responsible for substantial yield losses in open field as well as in greenhouse tomato production (for a review, see McGovern [29]). Diseased plants show wilting, stunting, and yellowing of leaves, which is caused by occlusion of discrete sectors of the vascular tissue and is accompanied by vascular discoloration. Chlamydospores remain viable in the soil for several years and remain an important inoculum source for root infection via the soil. To date, three races of *Fol* have been identified as well as the corresponding resistance genes [30]. However, not all cultivars carry these resistance genes, and landraces or heirloom cultivars might be of specific interest for organic production or for other traits, such as abiotic stress tolerance [31,32]. The management of *Fusarium* wilt includes several strategies, such as cultivar selection and chemical, physical, and biological control measures. Biological control based on the use of beneficial microorganisms can offer alternatives for chemical treatments, but detailed knowledge of the underlying mechanisms is necessary for optimum results. This also includes studies at the cultivar level.

To date, little is known about specific interactions between cultivars, endophytic fungi, and pathogens, and there is a special focus on induced systemic resistance and increased disease tolerance in tomato. For instance, Steinkellner, et al. [33] showed that tomato cultivars differed in their response to *Fol* depending on AMF inoculation. Furthermore, cultivar-specific reactions of tomato were reported after resistance induction through rhizobacteria or silicon application, expressed in modifications of the plant cell wall and expression of defense-related enzymes [34,35]. Moreover, there is also evidence of cultivar-specific differences in growth promotion triggered by *S. indica* in barley [5], indicating putative cultivar-specific effects of *Serendipita* spp. The role of cultivar-specificity in induced systemic resistance (ISR) and disease tolerance is a research gap that needs to be addressed in future breeding strategies as proposed for AMF [36]. Hence, identification of the most effective endophytic fungi for certain tomato cultivars could provide further evidence for such complex interactions and tools in a toolbox for integrated management strategies against *Fusarium* wilt.

Therefore, we aimed to investigate (i) the biocontrol potential of the two European isolates, *S. herbamans* and *S. williamsii*, against *Fol* in comparison to *S. indica* and *S. vermifera* in susceptible and tolerant tomato cultivars; (ii) the effects of *Serendipita* spp. on plant growth and development of the two cultivars; and (iii) direct inhibitory effects of *Serendipita* spp. on *Fol*.

## 2 Materials and Methods

### 2.1 Fungal Cultivation and Inoculum Production

*Serendipita indica* (DSM 11827), *S. williamsii* (DAR 29830), *S. herbamans* (DSM 27534), and *S. vermifera* (MAFF 305830) were cultivated on modified Käfer medium [37] or malt yeast peptone (MYP) medium at 24 °C in darkness for 3–4 weeks. For inoculum production, five plugs of 5 mm diameter from 4-week-old cultures were added to 500 mL of liquid Käfer medium for *S. indica* and *S. williamsii* and to liquid MYP medium for *S. herbamans* and *S. vermifera*. Subsequently, flasks were incubated on an orbital shaker at 24 °C in darkness for 2–3 weeks. Before inoculation, mycelium from liquid culture was homogenized for one minute in a high-speed blender. The suspension was centrifuged for 5 min at 10,000 rpm at 4 °C, and the pellet was washed with sterile dH_2_O to remove the media. The chlamydospores and/or mycelial fragments were counted using a Fuchs-Rosenthal counting chamber, and the concentration was adjusted to 3 × 10^5^ cfu mL^−1^ [15].

*F. oxysporum* f. sp. *lycopersici* (Sacc.) W.C. Snyder & H.N. Hansen (race 2, isolate 007), kindly provided by B.J. Cornelissen, (University of Amsterdam, The Netherlands), was cultivated on Czapek Dox (CzD) agar (Duchefa Biochemie, Haarlem, The Netherlands) and incubated at 24 °C in darkness for two weeks. For *Fol* inoculation, chlamydospores were prepared using a slight modification of the method described by Goyal, et al. [38] and Bennett and Davis [39]. Microconidia were harvested by flooding the two-week-old culture plates with dH_2_O and rubbing the surface with a Drigalski spatula. The suspension of conidia and hyphae was filtered through three layers of cheese cloth filter (20–150 μm pore diameter; Laporte Ges.m.b.H., Wels, Austria) to separate the conidia from hyphae. The conidial concentration was adjusted to 1 × 10^7^ microconidia mL^−1^ using a Thoma counting chamber. To prepare soil broth, potting soil (Aussaaterde, Gramoflor GmbH & Co. KG, Vechta, Germany) was autoclaved and mixed with autoclaved dH_2_O at a ratio of 1:4 (*w*/*v*); for this purpose, 250 g of soil was added to a volume of 1000 mL of autoclaved dH_2_O and agitated on an orbital shaker for 60 min at 90 rpm. The mixture was sieved through a 1.5 or 1 mm mesh sieve to remove coarse materials and then filtered through 8 layers of fleece cloth filter (150 μm). Afterwards, 0.025 g of glucose was added to each 50 mL of filtered soil broth and autoclaved at 121 °C for 20 min. The following day, the autoclaved soil broth was transferred to another bottle to reduce insoluble impurities or sediment at the bottom of the bottle and autoclaved for a second time. All remaining insoluble impurities were allowed to settle for a few hours. After sedimentation, 50 mL of the clear soil broth was decanted into individual 125 mL Erlenmeyer flasks. Each Erlenmeyer flask containing 50 mL of soil broth was inoculated with 300 μL of conidial suspension (1 × 10^7^ microconidia mL^−1^) and incubated near a window under natural light conditions at room temperature (24–27 °C) for 10 to 14 days to produce chlamydospores. Thereafter, the Erlenmeyer flask contents were homogenized in a high-speed blender, and chlamydospores were quantified using a Fuchs-Rosenthal counting chamber.

### 2.2 Plant Cultivation and Inoculation

Tomato seeds (*Solanum lycopersicum* L. cv. Kremser Perle and cv. Micro-Tom) were surface-sterilized by soaking in 50% household bleach (2.8% NaOCl *w*/*w*) for 10 min. The household bleach was washed away by replacing the solution with autoclaved ddH_2_O three times. The surface-sterilized tomato seeds were germinated in pots filled with autoclaved perlite (Granuperl S 3–6, Knauf Perlite GmbH, Vienna, Austria) and incubated in a growth chamber (Rumed, Rubarth Apparate GmbH, Germany) at 24 °C with a 14/10 h light/dark photoperiod (light intensity 296 *μ*mol m^−2^ s^−1^) for three to four weeks. After reaching the first true leaf stage, tomato seedlings were removed from the perlite. Root tips were clipped to facilitate the entry of inoculum. In the first step, roots were inoculated with suspensions of chlamydospores and/or mycelial fragments of selected *Serendipita* spp. at a concentration of 3 × 10^5^ cfu mL^−1^ [15] by submerging the roots for 24 h. Control plants were treated in a similar way as inoculated plants and were dipped in autoclaved dH_2_O. For *Fol* inoculation, prepared chlamydospore suspensions were mixed with soil at a final concentration of 5000 chlamydospores g^−1^ of substrate before transplanting the tomato seedlings.

Pots were filled with a mixture of soil (Aussaaterde, Gramoflor GmbH & Co. KG, Vechta, Germany), sand (Quarzsand 0–3 mm, Quarzwerke Österreich GmbH, Melk, Austria) and clay (Liapor fit 1–4 mm, Lias Österreich GmbH, Fehring, Austria) (1:1:1, *v*/*v*/*v*), and the tomato seedlings were transplanted into the prepared pots. Plants were grown in a completely randomized design in a greenhouse and were watered regularly with tap water. Twice a week, plants received 50 mL of a nutrient solution consisting of the following components L^−1^: 3.47 mM Ca(NO_3_)_2_, 1.50 mM K_2_SO_4_, 0.0898 mM KH_2_PO_4_, 3.0715 mM MgSO_4_, 0.100 mM NH_4_NO_3_, 0,0988 mM Fe_6_H_5_O_7_ × 3 H_2_O, 0.0048 mM Na_2_Bo_4_O_7_ × 4 H_2_O, 0.0021 mM ZnSO_4_ × 7 H_2_O, 0.0018 mM CuSO_4_ × 5 H_2_O, 0.0038 mM MnCl_2_ × 4 H_2_O, and 0.0005 mM MoO_3_ [40]. The experimental setup consisted of the following 10 treatments: (i) control, (ii) *S. indica* (iii) *S. williamsii*, (iv) *S. herbamans*, and (v) *S. vermifera* with (+*Fol*) and without *Fol* (−*Fol*). Each treatment consisted of 12 replicate pots per time-independent trial. All experiments were run in two time-independent repetitions (trials) in a greenhouse at a day/night temperature of 24 °C/18 °C and relative humidity of 60%. Additional light was provided under a photoperiod of 16 h when outside photosynthetically active radiation (PAR) was below 367.43 μmol m^−2^ s^−1^. Experiments were conducted from early August to early October 2016 (trial I) and from the middle of August to the middle of October 2016 (trial II) with the cv. Micro-Tom as well as from September to November 2016 (trial I) and from February to April 2017 (trial II) with the cv. Kremser Perle. Temperature within the greenhouse cabin was recorded every 10 min and daily mean, maximum, and minimum temperatures are presented in Figure S1. After 8 weeks, plants were harvested, and the roots were gently washed under tap water. Afterwards, plants were assessed for the growth parameters, and the phenological development was recorded according to the extended BBCH-scale by using the codes for solaneous fruits [41].

Disease progression was visually evaluated using the scale of Wellman [42] for three sets of five randomly chosen plants per treatment and independent repetition (*n* = 6). In brief, the stems of tomato plants were split open with a scalpel and the progress of vessel discoloration within the stem together with leaf wilt symptoms were used for rating the plants from 0 (healthy) to 15 (dead). To allow weighting the impact on the host plant, ratings were assigned to five groups (*g*1 = 0.5, 1; *g*2 = 2; *g*3 = 3, 4; *g*4 = 5, 6; *g*5 = ≥7), and disease severity was calculated by using the following formula [43]:

(1) $$Disease\;severity=\frac{5\times \left(n_{g1}+2\;n_{g2}+5\;n_{g3}+10\;n_{g4}+20\;n_{g5}\right)}{\text{n}\;\text{diseased}\;\text{plants}}$$

The percentage of disease incidence was calculated by counting the number of infected plants relative to the total number of plants for three groups of four randomly chosen plants per treatment and independent repetition (*n* = 6). Additionally, *Fol* infection was confirmed by incubating a surface-sterilized piece of the hypocotyl (0.5 cm) on potato dextrose agar plates amended with streptomycin (10 mg L^−1^) at 24 °C in darkness followed by microscopic analysis of reisolated *Fol* morphological structures [33,44].

### 2.3 Detection of *Serendipita* spp. in Tomato Roots

To detect different *Serendipita* spp. in roots of both tomato cultivars, a polymerase chain reaction (PCR) was conducted using a primer pair specific for the *Serendipita* spp. translation elongation factor EF-1*α* gene (*PiTef*). Using the DNeasy Plant Mini Kit (Qiagen, Hilden, Germany), the total DNA was extracted according to the manufacturer’s instructions from ≤100 mg tomato roots sampled from the following variants 56 days after inoculation: Noninoculated control plants, plants inoculated with *Fol*, plants inoculated with different *Serendipita* spp. and plants inoculated with both *Fol* and different *Serendipita* spp. As a positive control, the DNA isolated from the mycelium of different *Serendipita* spp. was used. PCRs were performed with three randomly selected samples from each treatment. Each 15 μL reaction contained 1 μL of template DNA, 5 μL of ddH_2_O, 7.5 μL of 2× Green GoTaq Reaction Buffer (Promega, Madison, WI, USA), and 0.75 μL of forward and reverse primer (PiTeff: ATCGTCGCTGTCAACAAGAT, PiTefr: ACCGTCTTGGGGTTGTATCC modified after Deshmukh et al. [11]). The reactions were performed with an initial 3 min denaturation step at 94 °C, followed by 34 cycles at 94 °C for 40 s, 54 °C for 30 s, and 72 °C for 30 s, followed by a final extension at 72 °C for 2 min. The presence of amplified PCR products was confirmed by electrophoresis using a 2% agarose gel in 1× TAE buffer.

### 2.4 Antagonistic Activity Assay

The interaction between antagonist fungi and *Fol* was evaluated by the method described by Ghahfarokhi and Goltapeh [21] with slight modifications.

For this purpose, to evaluate the inhibition of *Fol* mycelium growth, one mycelial disc (5 mm) of each selected endophytic fungus from a 2-week-old culture was placed on one side of a PDA plate (9 cm), 2 cm away from the periphery and incubated at 24 °C in darkness for 7 days. After 7 days of incubation, the mycelial discs of *Fol* from a 2-week-old culture were also placed in a similar way but on the opposite side of the plate. As a control treatment, fresh PDA plates were inoculated with mycelial discs of *Fol* in a similar way. Both inoculated and control plates were incubated at 24 °C in darkness, and the percentage inhibition of radial growth (PIRG) was assessed by measuring the radial growth of *Fol* every day in both plates in the direction of the antagonist (R_2_) as well as the control plates (R_1_), until *Fol* covered the entire plates. Additionally, the growth rates of the endophytes were assessed. The data were calculated using the following formula [45]:

(2) $$PIRG=\frac{\left(R_{1}-R_{2}\right)\times 100}{R_{1}}$$

For each treatment, 12 replicates were considered, and the entire experiment was repeated 3 times.

### 2.5 Statistical Analysis

In the first step, biometric data (BBCH, shoot dry mass and root dry mass) of each tomato cultivar were analyzed for differences between the two independent repetitions by one-way ANOVA. Because differences between repetitions were significant for some parameters, biometric data were analyzed considering the factors *‘Serendipita’* and ‘trial’ for −*Fol* and +*Fol* treatments, separately. According to Levene’s test (*p* < 0.05), some biometric parameters did not meet the homogeneity of variance assumption and, to avoid data transformation, robust two-way ANOVA was used [46]. Analyses were conducted using the software RStudio 1.1.453 [47] and the ‘WRS2’ package ver. 1.0-0 [48]. Two-way analyses were conducted considering the factors *‘Serendipita’* and ‘trial’ and their interactions using the function pbad2way(), which calculates M-estimators for location based on medians (*p* < 0.05). The number of bootstrapping samples was set to 599. When the factors *‘Serendipita’*, ‘trial’ or the *‘Serendipita* × trial’ interactions were significant, post hoc comparisons based on M-estimators for location and bootstrapping were performed using the function mcp2a(). Contrasts were considered to be significant when confidence intervals for Huber’s $\hat{\Psi }$ did not cross zero [49]. Disease incidence and disease severity in cv. Kremser Perle was analysed by one-way ANOVA using the software IBM SPSS Statistics 24 (IBM, Armonk, NY USA). Mean separation was performed using Tukey’s test (*p* < 0.05). Disease ratings for Micro-Tom were analyzed by the nonparametric Kruskal Wallis test (*p* < 0.05) due to many ratings with 0. Figures were prepared with the software Sigma Plot 14.0 (Systat Software, Inc., San Jose, CA, USA). The obtained data for the antagonistic activity of *Serendipita* species against *Fol* were analyzed based on one-way ANOVA and Tukey’s post hoc test (*p* < 0.05).

## 3 Results

### 3.1 Effects of *Serendipita* spp. and *Fol* Inoculation on the Phenological Growth Stages of Tomato Plants

**Figure 1:**
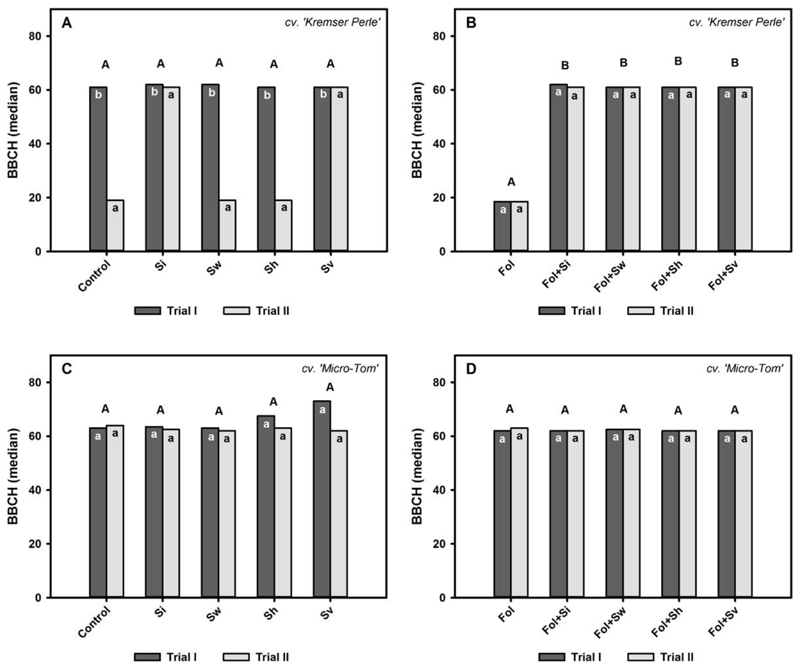
Median BBCH values for the cv. Kremser Perle and the cv. Micro-Tom treated with different *Serendipita* spp. (Si = *S. indica*, Sw = *S. williamsii*, Sh = *S. herbamans*, Sv = *S. vermifera*) without (**A**,**C**) and with (**B**,**D**) *F. oxysporum* f. sp. *lycopersici* (*Fol*) inoculation over trial I and trial II, 56 days after inoculation. Contrasts for the main factor ‘*Serendipita* spp.’ are indicated by upper case letters. Contrasts for the main factor ‘trial’ are indicated by lower case letters. Columns followed by the same letters are not significantly different (Wilcox robust post hoc test, *p* < 0.05, *n* = 12).

To reveal the effects of *Serendipita* spp. on tomato plant development, phenological growth stages were determined. The phenological growth stages of the cv. Kremser Perle without *Fol* application were significantly affected by the factor ‘trial’ (Table 1). Average (21.2 ± 0.1 °C and 20.9 ± 0.1 °C) as well as maximum daily temperatures (24.9 ± 0.3 °C and 24.1 ± 0.1 °C) differed between trial I and trial II of the cv. Kremser Perle, especially during early development (Figure S1A). In the first trial, the median for the BBCH rating for all treatments occurred during the flowering stage (BBCH 61 and 62, respectively) (Figure 1A). In the second trial, plants developed more slowly. Plants in the control, *S. williamsii* and *S. herbamans* treatments remained in the leaf development stage (BBCH 19), whereas plants in the *S. indica* and *S. vermifera* treatments reached the flowering stage (BBCH 61). However, this trend was not statistically significant (Table 1, Figure 1A). Under *Fol* disease stress, the factor *‘Serendipita’* significantly affected plant growth performance (Table 1). Plants in the +Fol treatment in both trials did not develop further until the leaf development stage (BBCH 18.5) (Figure 1B). The four *Serendipita* spp. in combination with *Fol* alleviated this effect and led to a significant increase in growth development until the flowering stage (BBCH 61 and 62, respectively). Micro-Tom plants were not affected by the factor *‘Serendipita’* or by the factor ‘trial’ in the −*Fol* and +*Fol* treatments, respectively (Table 1). Ratings ranged between flowering stages (BBCH 62) and fruit development stages (BBCH 73) in the −*Fol* treatments (Figure 1C). In the +*Fol* treatments, plants reached the flowering stages (BBCH 62 and 63) (Figure 1D). Micro-Tom trials were characterized by a high fluctuation in mean and maximum daily temperatures (Figure S1B). The mean maximum daily temperature was 28.7 ± 0.3 °C and 27.8 ± 0.3 °C in trial I and trial II, respectively. The average daily temperature was 23.3 ± 0.2 °C and 22.7 ± 0.2 °C, respectively.

### 3.2 Effects of *Serendipita* spp. and *Fol* Inoculation on Tomato Plant Biomass

**Figure 2:**
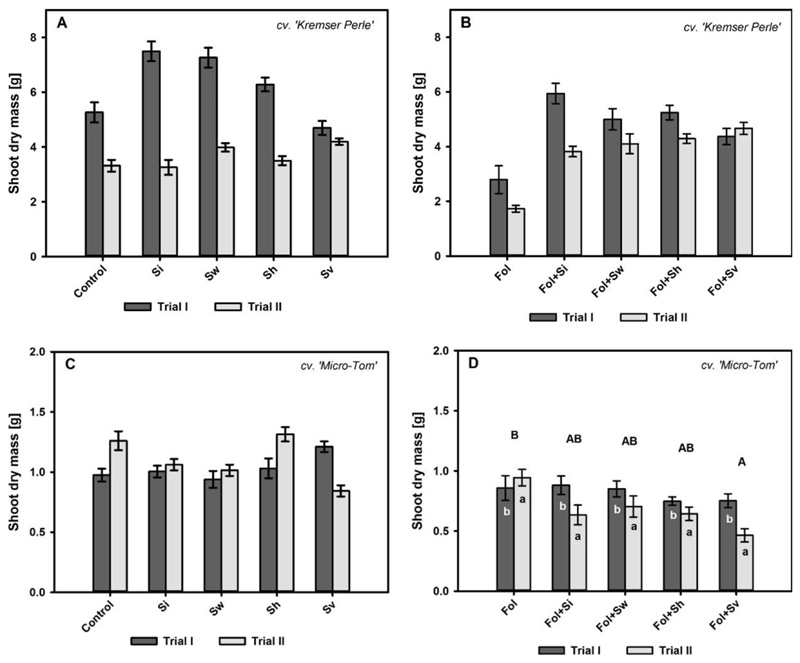
Shoot dry mass (mean ± S.E.) in g for the cv. Kremser Perle and cv. Micro-Tom treated with different *Serendipita* spp. (Si = S. *indica*, Sw = *S. williamsii*, Sh = *S. herbamans*, Sv = *S. vermifera*) without (**A**,**C**) and with (**B**,**D**) *F. oxysporum* f. sp. *lycopersici* (Fol) inoculation over trial I and trial II (56 days after inoculation). Contrasts for the main factor ‘*Serendipita*’ are indicated by upper case letters. Contrasts for the main factor ‘trial’ are indicated by lower case letters. Columns followed by the same letters are not significantly different (Wilcox robust post hoc test, *p* < 0.05, *n* = 12). When main effects could not be interpreted due to interaction effects, contrasts for significant ‘*Serendipita* × trial’ interactions are presented within the text of the results section.

The shoot dry mass of the cv. Kremser Perle was significantly affected by the *‘Serendipita* × trial’ interaction in −*Fol* (*p* < 0.0001) and +*Fol* (*p* < 0.05) treatments (Table 1). In the −*Fol* treatments, the first trial was characterized by a higher shoot dry mass development, i.e., 59%, compared to the second trial (Figure 2A). *‘Serendipita* × trial’ interaction effects were observed between the control and *S. indica* $\left(\hat{\Psi }=-1570,\;p<0.0001\right)$ and *S. williamsii* $\left(\hat{\Psi }=-1.050,\;p<0.01\right)$ treatments, showing an increase of shoot dry mass of 42% and 38%, respectively, in the first trial. Another source of interaction wes observed between *S. vermifera* and *S. indica* $\left(\hat{\Psi }=4.185,\;p<0.0001\right),$ *S. williamsii* $\left(\hat{\Psi }=-2.890,\;p<0.0001\right)$ and *S. herbamans* $\left(\hat{\Psi }=2.865,\;p<0.0001\right),$ which was mainly characterized by a stable development of the shoot dry mass in *S. veomifera* treatments with 4.7 and 4.2 g, respectively, over the two trials compared to *S. indica, S. williamsii*, and *S. herbamans* treatments.

The shoot dry mess of the cv. Kremser Perle was significantly affected by the *‘Serendipita* × trial’ interaction in +*Fol* treatments (*p* < 0.05) (Table 1, Figure 2B). *Fol* reduced the shoot dry mass to 2.79 and 1.73 g in trials I and II, respectively (Figure 2B). All *Serendipita* spp. treatments increased the shoot dry mass by between 57% and 170% compared to the +*Fol* treatment. Significant *‘Serendipita* × trial’ interactions occurred between *S. indica* and *S. vermifera* $\left(\hat{\Psi }=2.609,\;p<0.05\right),$ mainly characterized by a stable development of *S. vermifera* over the two trials (4.37 and 4.67 g, respectively).

For the cv. Micro-Tom, shoot dry mass was significantly affected by a *‘Serendipita* × trial’ interaction in the −*Fol* treatments (Table 1, Figure 2C). In the second trial, the shoot dry mass of the control treatment was increased by 29% compared to the first trial (Figure 2C). A similar pattern was observed for the *S. herbamans* treatment. This is in contrast to the *S. indica* treatment $\left(\hat{\Psi }=-0.235,\;p<0.0001\right),$ where the shoot dry mass with 1.01 and 1.06 g, respectively, was similar over the two trials. Furthermore, the *S. vermifera* treatment showed an increase in shoot dry mass in trial I whereas there was a reduction in shoot dry mass in trial II compared to the control $\left(\hat{\Psi }=-0.645,\;p<0.0001\right)$ and the *S. herbamans* treatment $\left(\hat{\Psi }=-0.690,\;p<0.0001\right),$ respectively.

The shoot dry mass of the cv. Micro-Tom was significantly affected by the factors *‘Serendipita’* and ‘trial’ under *Fol* disease stress (Table 1, Figure 2D). Shoot dry weights in the *Fol* treatments were significantly lower in trial I than in trial II $\left(\hat{\Psi }=0.545,\;p<0.05\right)$ (Figure 2D). Furthermore, the application of *S. vermifera* reduced the shoot dry mass by 32% compared to the +*Fol* control treatment over the two trials $\left(\hat{\Psi }=-0.410,\;p<0.05\right)$.

**Figure 3:**
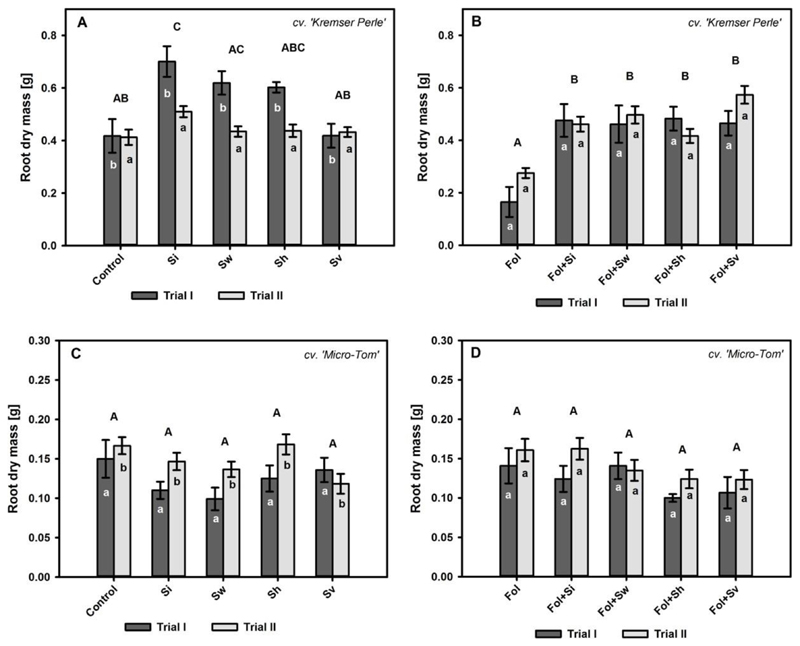
Root dry mass (mean ± S.E.) in g for the cv. Kremser Perle and cv. Micro-Tom treated with different *Serendipita* spp. (Si = *S. indica*, Sw = *williamsii*, Sh = *S. herbamans*, Sv = *S. vermifera*) without (**A**,**C**) and with (**B**,**D**) *F. oxysporum* f. sp. *lycopersici* (*Fol*) inoculation over trial I and trial II (56 days after inoculation). Contrasts for the main factor ‘*Serendipita*’ are indicated by upper case letters. Contrasts for the main factor ‘trial’ are indicated by lower case letters. Columns followed by the same letters are not significantly different (Wilcox robust post hoc test, *p* < 0.05, *n* = 12).

The root dry mass of the cv. Kremser Perle in thp −*Fol* treatments was significantly affected by the main factors *‘Sertndipita’* (*p* < 0.01) and ‘trial’ (*p* < 0.0001) (Table 1, Figure 3A). Trial I was characterized by a higher root dry mass development than trial II $\left(\hat{\Psi }=0.528,\;p<0.0001\right).$ Furthermore, a significant increase in the root dry mass of the *S. indica* treatment (0.61 g) compared to the control (0.42 g) $\left(\hat{\Psi }=-0.37500,\;p<0.01\right)$ and *S. vermifera* treatment (0.43 g) $\left(\hat{\Psi }=0.440,\;p<0.0001\right)$ was evident. The root dry mass of tha cv. Kremser Perle in the +*Fol* treatments was significantly affected by the factor *‘Serendipita’* (*p* < 0.0001) (Table 1, Figure 3B). In the +*Fol* treatments, the application of *Serendipita* spp. increased root dry weights by 105 to 136% compared to +*Fol* alone.

The root dry mass of the cv. Micro-Tom in the −*Fol* treatments differed significantly over the two trials $\left(\hat{\Psi }=-0.135,\;p<0.05\right),$ but for the main effect, *‘Serendipita’* post hoc procedures did not reveal any significant differences (Table 1, Figure 3C). *Fol* application did not have an effect, alone or in combination with *Serendipita* spp., on the root dry mass of Migro-Tom plants (Table 1, Figure 3D).

### 3.3 *Fol* Disease Incidence and Severity in Planta

To evaluate the effects of selected *Serendipita* spp. on plant resistance to *Fusarium* wilt, disease incidence and severity were scored eight weeks after pathogen inoculation for each tomato cultivar. With respect to the cv. Kremser Perle, the application of *Serendipita* spp. had a significant (*F*_(4,25)_ = 7.597, *p* < 0.0001) effect on the disease incidence (Table 2). The disease incidence was significantly reduced from 93 ± 4% in the *Fol* treatment to 47 ± 7% and 48 ± 7% when coinoculated with *S. herbamans* and *S. vermifera*, respectively. Inoculation with *S. indica* (87 ± 10%) and *S. williamsii* (64 ± 9%) did not decrease disease incidence significantly. However, disease severity was not affected by the application of *Serendipita* spp. [*F*_(4,25)_ = 1.590, *p* = 0.208] (Table 2).

Disease incidence in the tolerant cv. Micro-Tom reached 20 ± 7% when *Fol* was applied alone and 18 ± 7% when *S. vermifera* was coinoculated (Table 2). The disease incidence in the treatments for *S. indica, S. williamsii*, and *S. herbamans* was reduced by 3, 7, and 0%, respectively. However, this reduction was not statistically significant. In all experiments, the −*Fol* treatments did not show any disease symptoms, and reisolation procedures did not show any *Fol* outgrowth.

### 3.4 Detection of Endophytic Fungi in Tomato Roots

**Figure 4:**
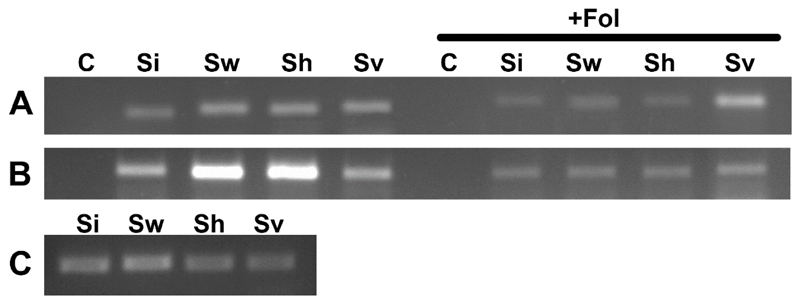
Polymerase chain reaction (PCR) detection of *Serendipita* spp. in tomato roots. Gel electrophoresis of PCR products amplified with the primer pair (PiTeff and PiTefr) specific for *Serendipita* spp. *PiTef* gene. The PCRs were performed with the genomic DNA extracted from tomato roots inoculated with selected endophytic fungi (Si, Sw, Sh, Sv) without *Fol* and with *Fol* as well as untreated plants and plants inoculated only with *Fol* as negative controls (**C**). A, cv. Kremser Perle; B, cv. Micro-Tom; C: Positive controls, PCR products amplified with DNA isolated from mycelia of selected endophytic fungi. C = control; Si = *Serendipita indica*; Sw = *S. williamsii*; Sh = *S. herbamans*; Sv = *S. vermifera*; Fol = *F. oxysporum* f. sp. *lycopersici*.

To detect different endophytic fungi in the tomato roots, PCR analysis was performed using a primer pair specific for the *Serendipita* spp. translation elongation factor EF-1*α* (*PiTef*) gene that is widely conserved among species and has been shown to be a useful tool for screening root samples for the presence of different *Serendipita* spp. [11]. PCR with the DNA isolated from roots of plants inoculated with all selected endophytic fungi ±*Fol* resulted in amplification of a specific 200 bp product (Figure 4A,B). This PCR product was not detected in control plants, which were inoculated only with *Fol* or were untreated (Figure 4A,B). All reactions performed with DNA isolated from the mycelia of selected endophytic fungi resulted in the amplification of the specific PCR product (Figure 4C).

### 3.5 Antagonistic Activity Assay against *Fol*

**Figure 5:**
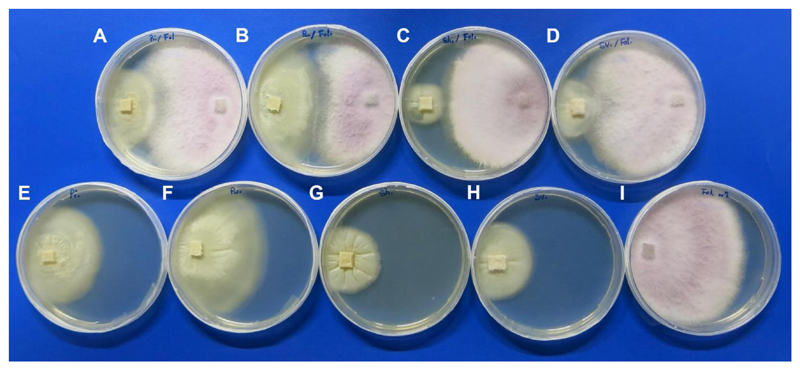
In vitro assay on antifungal activity of (**A**) *S. indica*, (**B**) *S. williamsii*, (**C**) *S. herbamans*, and (**D**) *S. vermifera* against *F. oxysporum* f. sp. *lycopersici* (*Fol*) after 9 days of confrontation on PDA plates and control plates with *S. indica* (**E**), *S. williamsii* (**F**), *S. herbamans* (**G**), *S. vermifera* (**H**), and *Fol* (**I**).

In vitro confrontation assays showed that *Fol* grew rapidly towards the *Serendipita* spp. colonies. In the treatments *S. indica* and *S. williamsii*, a contact zone could be observed (Figure 5); however, *Fol* passed this zone and was able continue its growth on the respective *Serendipita* spp. colonies. Due to the lack of inhibitory effects, PIRG could not be calculated. Phase contrast microscopy of these interaction zones revealed that *Fol* continued its growth next to the mycelia of the respective *Serendipita* spp. (Figure S2). Furthermore, neither hyphal coiling on *Fol* hyphae nor lysis of *Fol* hyphae was observed at 6 and 7 dpi. The mycelium of *Fol* growing on *S. herbamans* and *S. vermifera* colonies was more compact (Figure S3C,D) compared to the mycelium growing on *S. indica* and *S. williamsii* colonies (Figure S3A,B). Due to overgrowth of the *Serendipita* spp. colonies by *Fol*, PIRG was not calculated.

## 4 Discussion

Here, we report the effects of four different *Serendipita* spp. on two tomato cultivars differing in their susceptibility to *Fol*. It could be shown that, for the susceptible cv. Kremser Perle, the negative effects of *Fol* on phenological growth were alleviated by all four applied *Serendipita* spp. Furthermore, negative effects on shoot and root dry mass were compensated by between 57% and 170% and 105% and 136%, respectively. Apart from these similar effects in biometric parameters, the effects on disease incidence differed. Hence, *S. herbamans* and *S. vermifera* reduced the disease incidence from 93 ± 4% in the *Fol* treatment to 47 ± 7% and 48 ± 7%, respectively. For the treatments *S. indica* and *S. williamsii*, the reduction of disease incidence to 84% and 64%, respectively, was not significant. Based on these findings, we speculate that the mode of action differs between the applied *Serendipita* spp.

### 4.1 In Vitro Antagonistic Activity Assay against *Fol*

In the biological control of plant pathogens, direct modes of action such as antibiosis, competition, and parasitism and indirect ways of action via activation of host plant defenses can occur. In our in vitro confrontation assays on PDA, we addressed the question of whether the applied *Serendipita* spp. can directly affect *Fol;* however, none of the selected *Serendipita* spp. could suppress the growth of *Fol* in vitro. On the contrary, all *Serendipita* species were suppressed in their growth and *Fol* was able to overgrow *S. herbamans* and *S. vermifera* with a dense and *S. indica* and *S. williamsii* with a more sparse mycelium in vitro. In heat-treated soil, *Fol* forms a dense mycelial network on the root surface within two days after inoculation [50], and also in vitro *Fol* colonizes artificial medium quite rapidly. To date, successful biocontrol of *Fol* could for instance be achieved by inoculating a nonpathogenic *F. oxysporum* isolate that competes for nutrients on the rhizoplane [50] and induces resistance responses in the host plant [51,52]. In our experiments, all *Serendipita* spp. isolates appeared to be poor competitors for nutrients on PDA even when incubated seven days prior to the addition of *Fol. S. herbamans*, and *S. vermifera* grew very slowly on PDA and might have needed more incubation time prior to the addition of *Fol* in order to compete. Additionally, we could not observe evidence of inhibitory diffusible or volatile secondary metabolite production on PDA in vitro. This is in line with other studies [5,12] where *S. indica* did not show any inhibition of *F. culmorum* and *F. graminearum* in vitro but reduced the negative effects on the biomass of barley. Furthermore, barley plants inoculated with *S. indica* had higher ascorbate levels over a three-week post inoculation period [5], which might be responsible for the protective effect [13]. Only recently, it was shown that *S. vermifera* inhibits *B. sorokiniana* in vitro and in planta [23]. In vitro, signs of mycoparasitism such as hyphal coiling, penetration, and colonization of hyphae of *B. sorokiniana* were observed. Furthermore, the authors showed via transcriptomic analysis that *S. vermifera* reduced the virulence potential of the pathogen prior to host plant infection rather than causing extensive host transcriptional reprogramming [23]. These findings indicate different levels of complexity in such multitrophic interactions and might also be very specific with regard to pathogen–endophyte–plant combinations.

### 4.2 *Fol* Disease Incidence and Severity in Planta

In our experiments, plants were inoculated with *Serendipita* spp. via root dipping for 24 h and were then potted in substrate amended with *Fol* chlamydospores. Chlamydospores are the main source of *Fol* inoculum in the field and were selected to mimic the field situation. Evaluation of *Fol* disease incidence and severity was performed 56 days. In other studies, plants were cocultivated with *S. indica* for one to three weeks before plants were additionally challenged with different pathogens, showing a bioprotective effect in barley, tomato, and wheat [6,11–13,15]. This might also explain why *S. indica* did not reduce disease incidence in our study, although the negative effects on plant development were alleviated. However, studies on simultaneous and even delayed inoculation showed a reduction of disease development in maize, lentils, and wheat [6,22,53]. Similar effects could be observed for *S. vermifera* in lentils [22]. Another difference can be noted in the observed effects. In the presented studies, effects of the pathogen were alleviated by mitigating the loss of biomass and through the reduction of disease severity, but effects on disease incidence were not observed. Another important factor is the longevity of this bioprotective effect. The bioprotective effect could be transient by delaying disease development, as was also observed against *F. graminearum* in barley [12]. This could also have been the case for *S. indica* in our study where a putative protective effect in the beginning might have been transient. In contrast, the effects of *S. herbamans* and *S. vermifera* remained stable eight weeks after inoculation, indicating a superior performance to *S. indica* with the cv. Kremser Perle and *Fol*. Hence, the European isolate *S. herbamans* and the Australian isolate *S. vermifera* proved to be very effective against *Fusarium* wilt in the greenhouse. However, detailed studies are not available yet on whether host priming is involved and if so, the duration that plants stay primed and which defense pathways are involved. Additionally, transcriptomic studies on the endophyte–pathogen–plant level could give more insight on putative fungal–fungal interactions relevant for disease reduction in planta [23,54,55].

### 4.3 Effects of *Serendipita* spp. and *Fol* Inoculation on Tomato Plant Biomass

An often-mentioned trait of *S. indica* and *S. vermifera* is their role in growth promotion in many different plant species. Here, the main focus was on investigating the effects against *Fol*, but *Serendipita* spp. control treatments revealed that the effects depended on the fungal species, the environmental conditions, and the tomato cultivar. For Kremser Perle in the first trial, which was characterized by higher mean and maximum temperatures, especially during early development (Figure S1A), growth promotion effects of *S. indica* and *S. williamsii* were evident. This might be due to the different affinities of these two fungi with respect to the temperature and temperature-dependent growth rates of tomato plants [56].

Apart from different effects on growth promotion, the effects on the cv. Kremser Perle under *Fol* disease stress remained stable over the trials. For the cv. Micro-Tom, shoot dry mass was significantly affected by a *‘Serendipita* × trial’ interaction in the –*Fol* treatments. In addition, the mean and maximum temperatures differed between the trials (Figure S1B). Effects in the –*Fol* treatments were variable, ranging from neutral to growth impairment (*S. indica*) and from a growth increase to growth impairment (*S. vermifera*). There is evidence that *S. indica* can also reduce tomato plant development depending on the inoculum concentration, tomato growth stage, and substrate composition [15]. However, how different temperatures affect the interactions of *Serendipita* spp. with their host plants is not known yet. Further studies on such temperature dynamics would be very relevant for putative applications in crop production. Apart from factors mentioned above, light intensities have been proven to affect the interaction between PepMV and tomato plants inoculated with *S. indica* [15]. Plants cultivated under low light intensities showed an increased content of PepMV particles compared to plants without *S. indica*. Under high light intensities, PepMV particles were even significantly reduced. In our trials, plants received irradiation either from natural light or from high pressure sodium lamps depending on the available natural light (PAR < 367.43 μmol m^−2^ s^−1^). Spectra of natural light fluctuate depending on factors such as weather conditions, time of day, and season [57] whereas spectral composition and intensity of artificial light remain constant. The irradiance spectrum has specific effects on plant responses such as secondary metabolite production and photosynthesis [57,58], which also impacts on the carbon content of cells. The amount of PAR provided by natural and artificial light varied between the two trials due to variation in day length, thereby eventually altering the carbon content of cells, which is also a crucial factor in the mutualism–parasitism continuum of AM symbiosis [59]. Whether and how nutrient dynamics in plants mediated by *Serendipita* spp. are responsible for these interactions remains to be elucidated but would be very relevant for putative applications in crop production.

Changes in nutrient dynamics might also be responsible for the reduced shoot dry mass of Micro-Tom plants inoculated with *S. vermifera* and challenged with *Fol*. The cultivar Micro-Tom does not harbor the I2 resistance gene but shows tolerance to *Fol* race 2 [60]. Tolerance can be associated with negative effects such as reduced growth and yield and is still quite unexplored [61]. In the case of the cv. Micro-Tom, plants inoculated with *Fol* grew slower in the beginning than the respective control plants; however, this effect was transient and could not be observed after 3 to 4 weeks (data not shown). Apart from the reduced shoot dry mass, no effects on the disease ratings occurred, which might indicate that plants prioritize the response to *Fusarium* wilt. Conversely, in *Nicotiana attenuata, S. vermifera* improved plant growth without increasing N or P contents, but this effect was accompanied by increased development of *Manduca sixta* larvae and reduced production of trypsin proteinase inhibitors [17]. Thus, how nutrient dynamics, defense responses, and the less explored mechanisms of disease tolerance interact with endophytic fungi such as *S. vermifera* are worth investigating in future studies, especially within the context of cultivar-specific growth response.

## 5 Conclusions

In conclusion, we show that all tested *Serendipita* species alleviated negative effects on plant development in the susceptible cultivar Kremser Perle, whereas *Fol* disease incidence was reduced only by *S. herbamans* and *S. vermifera*. Moreover, the shoot dry mass of the *Fol*-tolerant cv. Micro-Tom was negatively affected by *S. vermifera* under *Fol* disease stress. Observed differences between *Serendipita* spp. in their protective effects could be based on the involvement of different mechanisms such as induced resistance or growth compensation or variation in the longevity of the effect. Hence, it is pivotal to investigate cultivar-specific effects in pathogen–endophyte–plant interactions to prevent such adverse effects and to determine the most beneficial combinations. Furthermore, we are confident that it is worth investigating other *Serendipita* species and strains for their potential in plant disease control.

## Supplementary Material

Supplementary file

## Figures and Tables

**Table 1:** Results from Wilcox robust ANOVA (*p* = 0.05) for the factors *‘Serendipita’*, ‘trial ’ and *‘Serendipita* × trial’ interactions.

Treatment	Factor	BBCH Code ^1^	Shoot Dry Mass [g]	Root Dry Mass [g]
*−F. oxysporum* f. sp. *lycopersici*				
cv. Kremser Perle	*Serendipita*	0.0801	**<0.0001**	**<0.01**
	trial	**<0.0001** ^2^	**<0.0001**	**<0.0001**
	*Serendipita* × trial	0.0684	**<0.0001**	0.0601
cv. Micro-Tom	*Serendipita*	0.8331	**<0.05**	**<0.05**
	trial	0.0501	0.1235	**<0.05**
	*Serendipita* × trial	0.4007	**<0.0001**	0.2588

*+F. oxysporum* f. sp. *lycopersici*				
cv. Kremser Perle	*Serendipita*	**<0.01**	**<0.0001**	**<0.0001**
	trial	0.5643	**<0.0001**	0.4574
	*Serendipita* × trial	0.8464	**<0.05**	0.1252
cv. Micro-Tom	*Serendipita*	0.6745	**<0.05**	0.0551
	trial	0.6260	**<0.05**	0.0918
	*Serendipita* × trial	0.6795	0.3556	0.7780

1 BBCH code according to Feller et al. [41].

2 Significant values (*p* < 0.05) are highlighted in bold.

**Table 2:** Disease parameters (mean ± S.E.) 56 days after pathogen inoculation (*n* = 6). Mean values followed by the same letters are not significantly different.

Cultivar	Treatment^1^	Disease Incidence [%]		Disease Severity [%]	
Kremser Perle ^2^					
	*Fol*	93 ± 4	B	27 ± 4	A
	Si + *Fol*	87 ± 10	B	31 ± 3	A
	Sw + *Fo*l	64 ± 9	AB	31 ± 6	A
	Sh + *Fol*	47 ± 7	A	17 ± 3	A
	Sv + *Fol*	48 ± 7	A	28 ± 6	A
		*F*(4,25) = 7.597		*F*(4,25) = 1.590	
		*p* < 0.0001		*p* = 0.208	

Micro-Tom ^3^					
	*Fol*	20 ± 7	A	12 ± 5	A
	Si + *Fol*	3 ± 3	A	1 ± 1	A
	Sw + *Fol*	7 ± 4	A	1 ± 1	A
	Sh + *Fol*	0 ± 0	A	0 ± 0	A
	Sv + *Fol*	18 ± 6	A	4 ± 2	A

1 *Fol* = *F. oxysporum* f. sp. *lycopersici;* Si = *Serendipita indica;* Sw = *S. williamsii;* Sh = *S. herbamans;* Sv = *S. vermifera*

2 ANOVA, Tukey’s test (*p* < 0.05)

3 Kruskal Wallis test (*p* < 0.05).
