# Supplementary material for: Serendipita Species Trigger Cultivar-Specific Responses to Fusarium Wilt in Tomato
Source: Agronomy (Basel). Author manuscript; Available in PMC 2019 Dec 19. (PMC6923139; doi:10.3390/agronomy9100595)
Supplement: Supplementary file [file EMS85065-supplement-Supplementary_file.pdf]

agronomy

MDPI

Supplemental Material

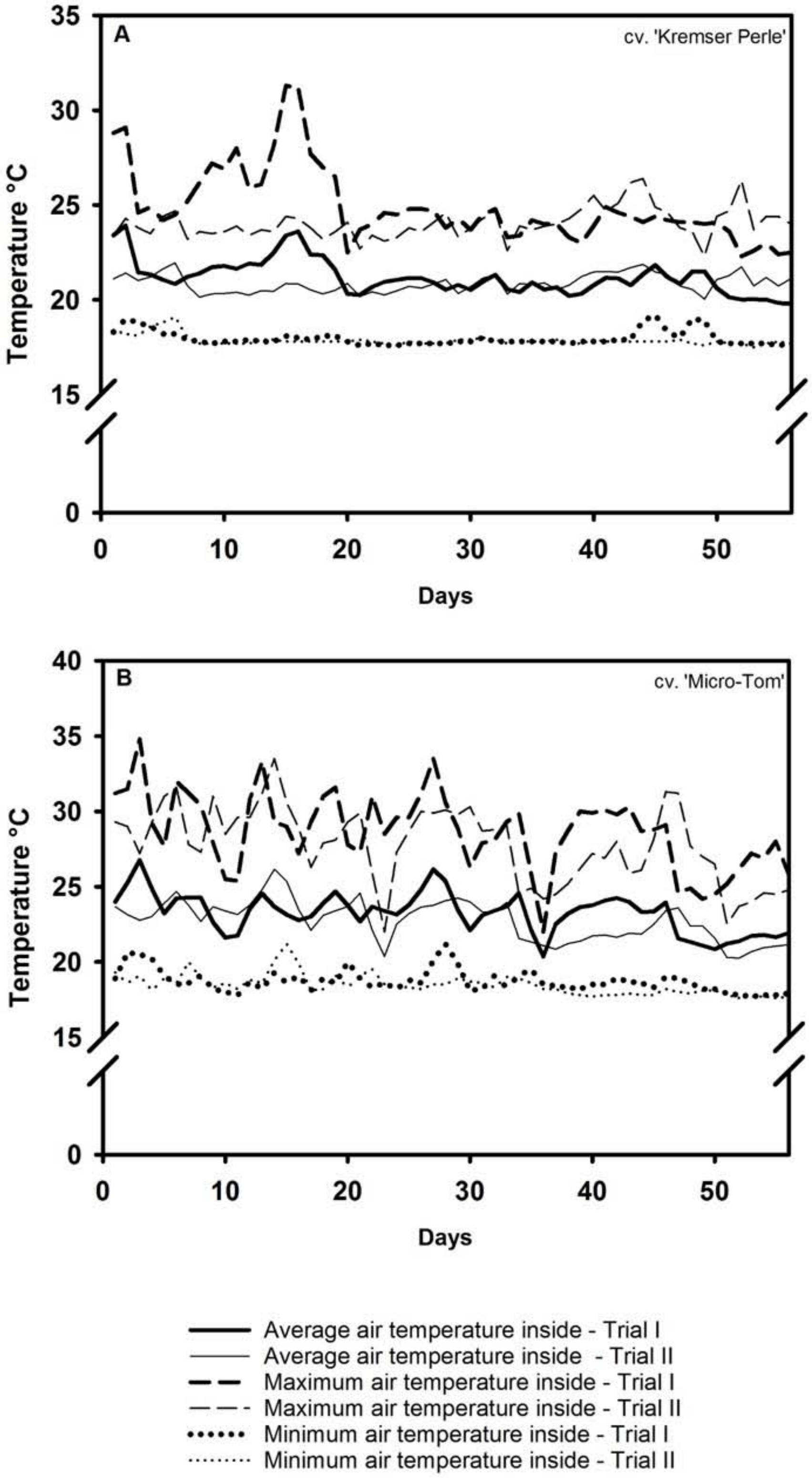

Figure S1. Average, maximum and minimum daily air temperature [°C] inside of the greenhouse cabin for the cv. Kremser Perle (A) and Micro-Tom (B) over trial I and trial II, respectively.

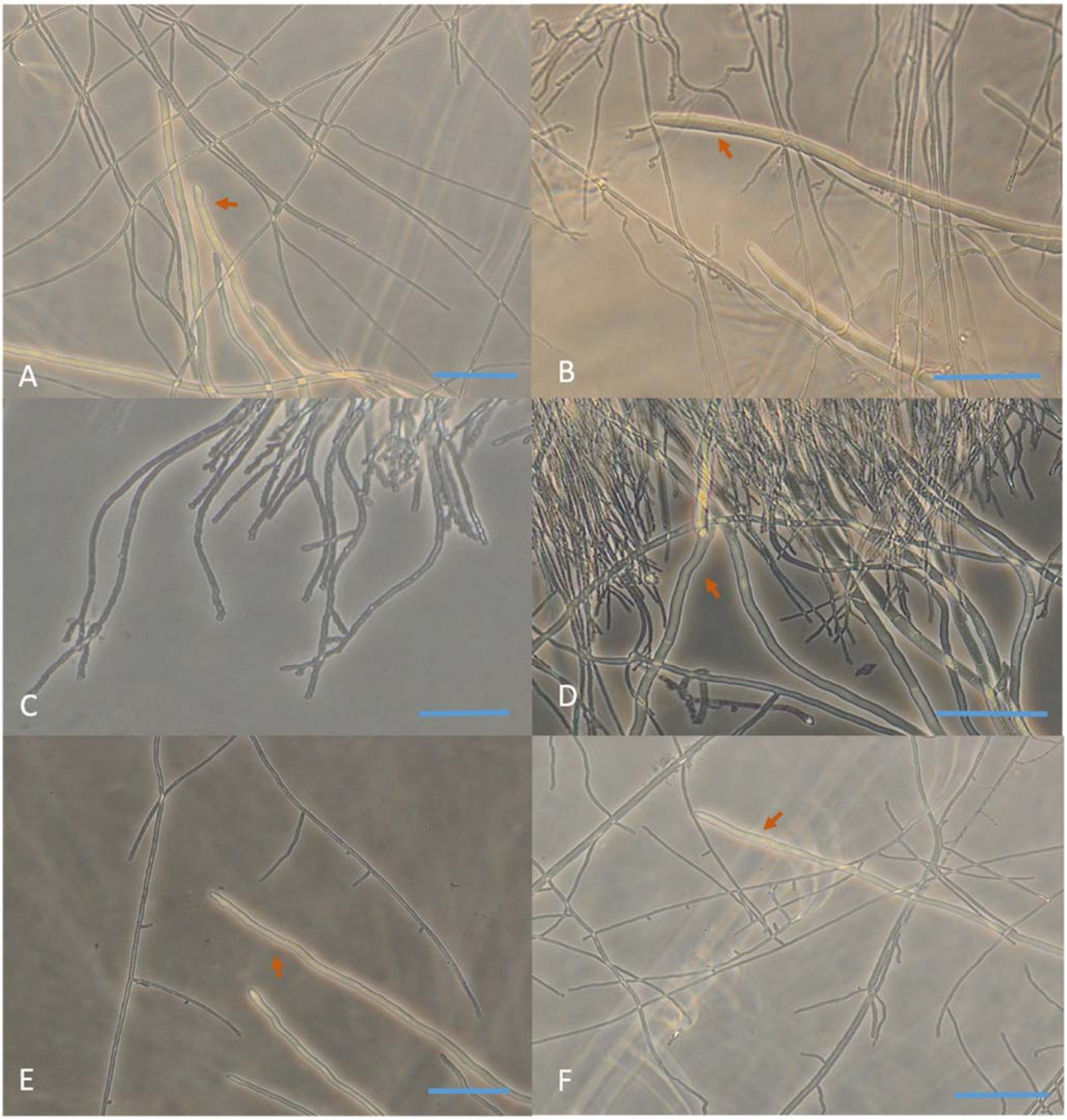

Figure S2. Interaction zone of *F. oxysporum* f. sp. *lycopersici* (*Fol*) with *S. indica* (A, 6 dpi), *S. williamsii* (B, 6 dpi), *S. herbamans* (D, 7 dpi) and *S. vermifera* (E, F, 7 dpi) on PDA. Hyphae of *S. herbamans* (C) on PDA (7dpi). Phase contrast. Arrows indicate hyphae of *Fol*. Scale bar: 50 μm (A, B, D, E, F), 20 μm (C).

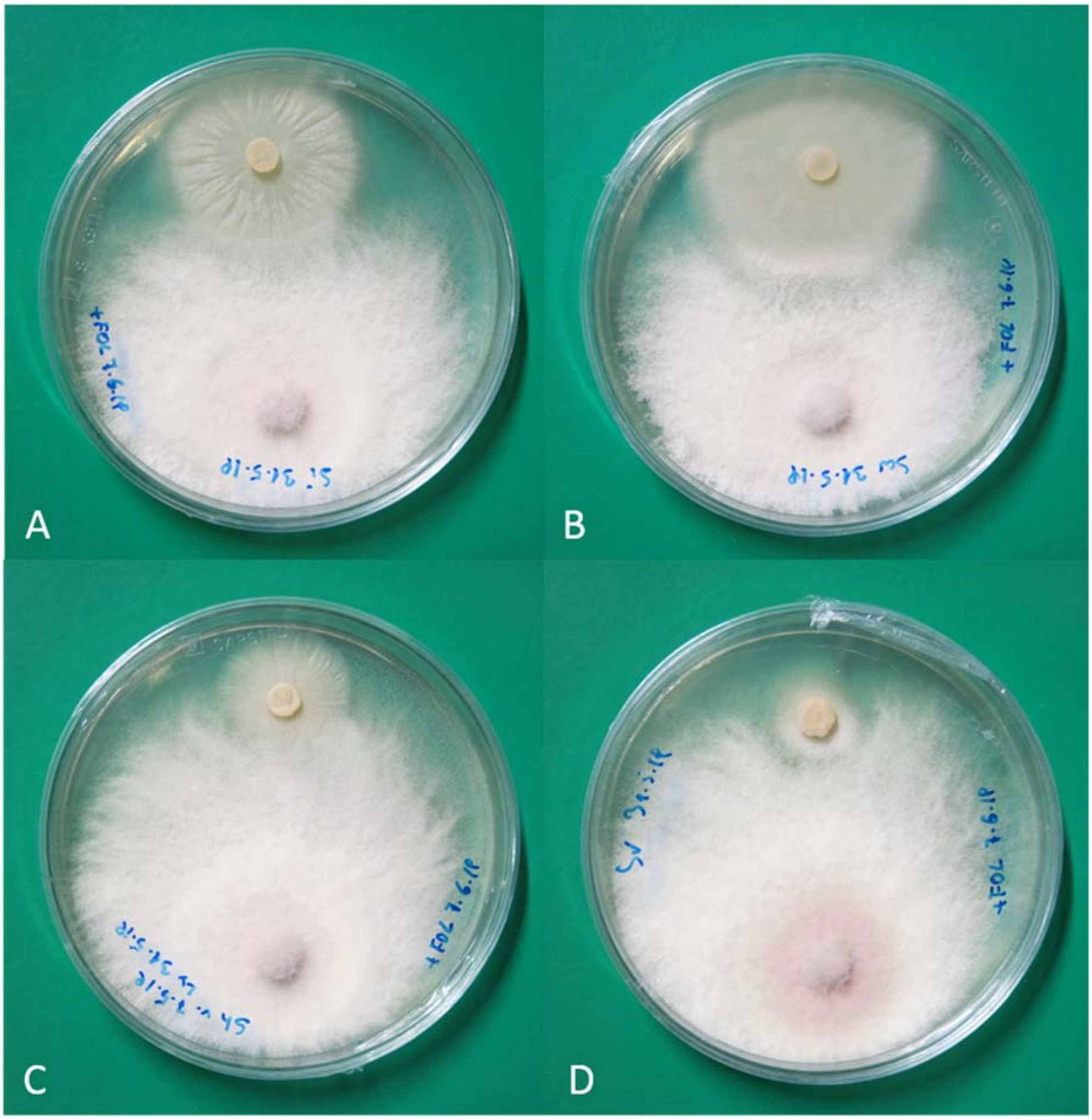

Figure S3. *In vitro* antifungal activity assay of (A) *S. indica*, (B) *S. williamsii*, (C) *S. herbamans* and (D) *S. vermifera* against *F. oxysporum* f. sp. *lycopersici* (*Fol*) 14 days after inoculation on PDA plates.
